# Clinical Discordance in Monozygotic Twins With Autism Spectrum Disorder

**DOI:** 10.7759/cureus.24813

**Published:** 2022-05-07

**Authors:** Angela Ho, Atif Towheed, Sandy Luong, Samuel Zucker, Eric Fethke

**Affiliations:** 1 Medicine, Touro College of Osteopathic Medicine, Middletown, USA; 2 Pediatrics, Touro College of Osteopathic Medicine, Middletown, USA; 3 Pediatrics, Drake University, Des Moines, USA; 4 Pediatric Cardiology, Boston Children's Hospital, Boston, USA

**Keywords:** unexplained syncope, attention-deficit/ hyperactivity disorder, autism spectrum disorder (asd), identical twins, monozygotic twins

## Abstract

There is a significant concordance of autism spectrum disorder in monozygotic (MZ) twins, where behavioral manifestations are heavily influenced by genetic factors. We describe a case of male monozygotic twins with autism spectrum disorder (ASD), raised in the same household, that present with different clinical manifestations. One of the twins presents with intermittent frank syncopal episodes, sinus bradycardia, and elevated alkaline phosphatase (ALP), while the other has symptoms of attention-deficit/hyperactivity disorder (ADHD), normal cardiological findings, and normal ALP level. The clinical discordance in this pair of monozygotic twins may be due to any of the following: 1) neuroanatomic cerebellar differences, 2) variable expression of genotype, and 3) inconsistent neurotransmitter regulation.

## Introduction

According to the Diagnostic and Statistical Manual of Mental Disorders (DSM), autism spectrum disorder (ASD) is characterized by the following: difficulty with social interactions, restricted interests, repetitive behaviors, and symptoms that interfere with one's ability to function independently in society. Early signs of autism can be seen as early as 12 months, including no babbling, no pointing, or regression in normal language. Some common features of ASD include hypersensitivity to five senses such as touch, smell, sounds, temperature, and taste [1]. Studies have suggested that environmental causes (i.e., toxins, social isolation) and genetic causes (i.e., synapse-related genes, gamma-aminobutyric acid (GABA)/ glutamate system dysregulation, neurodevelopmental genes) can contribute to the manifestation of ASD [2-4]. 

The concordance of ASD among monozygotic (MZ) twins ranges from 36% to 95% [5]. Social and repetitive behavioral manifestations of ASD are highly influenced by genetic factors [6]. Marked clinical variation among MZ twins has yet to be explained. MZ twins are genetically identical; however, clinical discordance has been reported in nine MZ twins, especially among males [7]. Variation in the clinical severity of ASD has been observed; however, the difference among MZ twins exhibiting a broad range of contrasts occurs rarely. Even when differences exist, both twins typically display levels of symptom burden [8]. In addition, phenotypic differences are noted between preterm versus at term gestation among individuals with ASD, such that individuals born preterm had higher incidences of sleep apnea, seizure disorders, and attention-deficit/hyperactivity disorder ADHD [9]. 

We report a case of MZ twins diagnosed with ASD that display variations in their clinical presentation. One twin presents with intermittent frank syncopal episodes and sinus bradycardia, while his twin presents with symptoms of ADHD but no evidence of pathological cardiological symptoms.

## Case presentation

Patient 1 and patient 2 are monozygotic twins who were born via Cesarean section at 36 weeks gestation and diagnosed with ASD at different ages. Patient 1 was diagnosed with ASD at three years of age, while patient 2 was diagnosed at six years of age. Both siblings are heterozygous for the MTHFR gene (OMIM 607093) A1298C variant (one copy) but otherwise revealed a normal genotype. They had no significant family history of seizures or cardiac pathologies. Both twins live in a special care facility.

Patient 1

The patient is a 13-year-old male with a past medical history of ASD, pervasive developmental delay, and cyclic neutropenia. Medications include daily intake of a multivitamin with fluoride, vitamin D 1000 IU, loratadine 10mg daily, and polyethylene glycol 10mg daily. He presented to the clinic with non-exertional syncopal episodes with seizure-like activity. This most recently witnessed syncopal episode was the third episode which occurred at approximately 1:30 am. The recovery lasted approximately nine minutes per a staff member of the special care facility.

On the physical exam, the patient was pleasant and in no apparent distress. His vitals were: blood pressure (BP) of 86/60, the temperature of 97.7° F, heart rate (HR) of 60 bpm, and oxygen saturation (SpO_2_) of 97%. His heart exam revealed a regular rhythm and rate, S1 and S2 sounds were present, and no indication of murmur, rub, gallops or clicks. His lungs were clear to auscultation bilaterally. His sinus bradycardia was at 60 bpm, consistent with a prior medical history of chronic bradycardia. 

The patient had several prior non-exertional syncopal episodes, with the most recent episode occurring seven and a half weeks prior to this clinic visit. The episode was notable for 90-seconds of staring with drooling as he slumped into his chair without bladder or bowel incontinence. There were no overt abnormal movements, and the patient recovered spontaneously with indications of lethargy. He was rapidly transferred to the local emergency department for further evaluation, where he presented with sinus bradycardia. A repeat electrocardiogram (ECG) twelve days after the initial diagnosis of sinus bradycardia showed normal sinus rhythm with sinus arrhythmia at 53 bpm, normal axis deviation of +80, QRS duration, ST and T wave segments were within normal limits. Three weeks prior to the second syncopal episode, the patient completed a video electroencephalogram (EEG), which was negative for seizure activity. Cardiac event monitoring and an echocardiogram were unremarkable. Basal metabolic panel and complete blood count were within normal limits, except for alkaline phosphatase (ALP), which was elevated (498 U/L). 

Patient 2

The patient is a 13-year-old male with a past medical history of ASD and attention-deficit/hyperactivity disorder (ADHD) presenting to the office for a routine monthly follow-up visit. Currently, there is no evidence of cardiorespiratory symptoms or adverse medication events. His medication history is significant for clonidine 0.2 mg twice per day for ADHD and quetiapine 25 mg per day for psychotic episodic symptoms. On physical exam, the patient was pleasant and in no apparent distress. His vitals were BP of 120/93, the temperature of 97.6°F, HR of 72 bpm, and SpO_2_ of 97%. His ECG showed normal sinus rhythm with sinus arrhythmia at 90 bpm, NAD of +45 degrees, and ST and T waves segments were within normal limits. There was no evidence of cardiotoxicity or innate tendency towards deleterious dysrhythmia. Basal metabolic panel and complete blood count were within normal limits.

## Discussion

ASD is associated with several diseases, such as epilepsy and congenital heart disease [10,11]. Patient 1 presented with syncope; however, the EEG was negative for seizure-like activity. Among several causes of syncope, vasovagal is one of the most common [12]. Patient 1's intermittent episodes of syncope may be vasovagal or neurological-related. Though patient 1's EEG was normal, it does not exclude epilepsy, as an EEG done several hours after a seizure can appear unremarkable [13]. Bhalla et al. reported an atypical case of syncope in children where one teenage boy's recurrent syncopal episodes were always preceded by an unintentional Valsalva maneuver while stretching [12]. A phenomenon described as "the fainting lark", a self-induced Valsalva-like maneuver that leads to acute cerebral hypoxia and syncope, has been reported as a compulsive behavior in other adolescents with ASD [14]. It is possible that Patient 1's syncopal episodes are similarly due to unintentional maneuvers. 

Furthermore, heart rate variability (HRV), a measure of cardiac autonomic function, when reduced, is typically observed in children with ASD [15]. This indicates an overall elevated average heart rate. Paradoxically, we observed sinus bradycardia in Patient 1. Studies suggest an autonomic imbalance that may contribute to the dysregulation of attentional and behavioral responses to external stimuli [15,16]. 

The twins demonstrate distinct behavioral differences. Patient 2 has been diagnosed with ADHD, while Patient 1 displays difficulty sitting still and focusing but does not meet the full DSM 5 criteria for ADHD. Preterm twins were found to have higher reports of ADHD, which is often noted as a common risk factor for both ASD and ADHD [9]. Patients 1 and 2 were born at 36 weeks of gestation, which may have contributed to the manifestation of their hyperactivity. However, the discordance in hyperactivity can be attributed to differing levels of dopamine and serotonin in the underlying pathophysiology of ASD between twins [17]. Patient 2's hyperactive symptoms have been well-controlled with quetiapine, while Patient 1 is not treated with atypical antipsychotics given his differing behavioral presentation. Although the mechanism of action of quetiapine is not fully understood, its effectiveness in treating patient 2's hyperactivity may be attributable to his dopamine and serotonin levels, unlike his brother's. 

Moreover, ALP was noted to be elevated in Patient 1, while Patient 2's levels are within normal limits. Elevated serum ALP is a marker for vitamin D deficiency, for which Patient 1 has been taking vitamin D supplements. Vitamin D serves as a neuroprotective factor in its neuronal calcium regulation, neurotransmitter regulation, and antioxidant activity [18]. Thus, the differing levels of ALP and vitamin D in the twins throughout their gestation or development may also contribute to the variance in behavioral features of ASD [18,19]. 

The variability in the clinical manifestations of ASD between Patient 1 and patient 2 may also be due to neuroanatomic discordance. Neural substrates within the brain structure have been hypothesized to be a potential cause of worsening autistic symptomatology severity [10]. Reports of twin pair differences in cerebellar anatomy indicate that such anatomic differences may be an important contributing feature between genetic liability and behavioral expressions [9]. Within discordant twins, children had lower cerebral white matter volumes in frontal and temporal lobes, which affect executive function, cognitive processing, and language that distinguishes children with ASD. Although there is no clear reason for autism-specific low volume related to limited white matter, it has been postulated that autism is associated with impairment of structural connectivity between particular brain zones [7]. The monozygotic twins in our report thus contribute additional evidence of variable ASD phenotypes despite identical genotypes [20]. 

## Conclusions

Although monozygotic twins have identical genes, twins with ASD can present with different phenotypes due to reasons, including variations in gene expression, shared versus non-shared environmental factors, and/or neuroanatomic differences. Phenotypic variations in the presentation of ASD between twins are a rare occurrence, which could also be attributed to limited reporting in the literature. Even though we present one case of twins, it is pertinent to identify such differences to customize treatment plans for each individual patient. Our understanding of the cause is severely limited. Further in-depth longitudinal studies need to be undertaken to uncover the etiology of such phenotypic differences. 
